# Musculoskeletal Pain and Satisfaction with an Injury-Prevention Workshop Among Young String and Wind Musicians: An Observational Study

**DOI:** 10.3390/healthcare14162498

**Published:** 2026-08-11

**Authors:** Clara Rodríguez-Gude, María Teresa Rocío Rial-Rodríguez, Lorenzo Antonio Justo-Cousiño

**Affiliations:** 1 Functional Biology and Health Sciences Department, Faculty of Physiotherapy, Universidade de Vigo, Campus a Xunqueira, s/n, 36005 Pontevedra, Spain; lorenzo.justo@uvigo.es; 2 Clinical Physiotherapy Research Group (FS1), Galicia Sur Health Research Institute (IIS Galicia Sur), SERGAS-UVIGO, 36312 Vigo, Spain; 3 Centro de Salud Taboada Leal, Servizo Galego de Saúde (Sergas), 36204 Vigo, Spain; maria.teresa.del.roc.rial.rodriguez@sergas.es

**Keywords:** back pain, behavior change, health education, musculoskeletal pain, musician, prevention

## Abstract

**Highlights:**

**What are the main findings?**

- Young musicians consider training on the prevention of musculoskeletal injuries to be important and interesting.
- The teacher is the main source of information about musculoskeletal injuries.

**What are the implications of the main findings?**

- Interventions aimed at raising awareness of musculoskeletal problems in musicians are key to increasing awareness and promoting their prevention.
- Physiotherapy plays an important role as a therapeutic option for both the treatment and prevention of this type of injury.

**Abstract:**

**Background/Objectives**: Musculoskeletal injuries are highly prevalent among musicians, with the neck and shoulders being the most frequently reported areas of pain. Preventive education should be included to help understand and avoid health risks associated with musical performance. The objective is to assess the level of satisfaction with a workshop on injury prevention among string and wind musicians, and to determine the prevalence and location of their musculoskeletal pain. **Methods**: This was an observational study consisting of the administration of a questionnaire following a workshop on musculoskeletal injury prevention. Inclusion criteria: string and wind musicians aged between 11 and 20 years. **Results**: A total of 83% were bowed string musicians. A total of 98.3% of participants indicated that the duration was appropriate, with a score of 8.9 for both overall satisfaction and perceived usefulness in instrumental practice. Thirty-five musicians reported musculoskeletal pain at some point in their lives, and it was present in 12 participants during the previous week. The most common pain locations were the left shoulder and left hand (45% and 40%, respectively), the cervical region (35%), and the right shoulder (33%). **Conclusions**: Musicians show a high level of satisfaction with the workshop received, considering it interesting and important for their instrumental practice. In addition, the participants expressed a high level of intention to carry out the proposed exercises. The prevalence of musculoskeletal pain is high, particularly in the shoulders, left hand, and cervical region.

## 1. Introduction

The practice of playing a musical instrument requires a high level of dedication and physical demand, which may lead to discomfort, functional alterations, and even the development of pathology due to overuse or chronic fatigue in specific muscle groups and joints [1,2,3]. In this context, tendon and muscle injuries resulting from prolonged postures and repetitive movements are widely recognized as occupational disorders among musicians [2,4]. Musculoskeletal disorders are highly prevalent in this population, with the neck and shoulder regions being the most frequently affected areas [5]. Key risk factors include repetitive movements, poor ergonomics, psychological stress, and the absence of preventive strategies [5].

Regarding knowledge and training, previous research has shown that higher music education curricula in Spain may include subjects related to body awareness or injury prevention. However, these are often optional, meaning that not all students are required to complete them as part of their training [6]. In contrast, at lower educational levels (elementary and professional), there is a lack of specific training focused on the body and its potential injuries. This occurs despite long-standing recognition of the role of educational institutions in addressing health-related issues and performance among performing arts students [7], as well as recommendations advocating the inclusion of preventive education to enhance understanding of and reduce health risks associated with musical performance [8,9].

Furthermore, injury prevention is considered a core component of physiotherapy practice, with health promotion and health education forming essential aspects of its professional scope [10]. Accordingly, physiotherapists with specialized training in performing arts or musicians’ health represent a valuable resource for delivering targeted educational interventions aimed at young musicians, thereby reducing the incidence of musculoskeletal disorders within this population.

On the other hand, participants’ perceived satisfaction is a relevant outcome in the evaluation of health education programs, as it may influence both the effectiveness of the intervention and the adoption of healthy behaviors. Previous studies have suggested that programs incorporating motivational components and providing a satisfying learning experience are more likely to promote positive changes in health-related habits and behaviors [11,12,13].

Based on the above, the primary aim of this observational study is to describe the level of satisfaction with an educational workshop on injury prevention among bowed string and wind musicians. Additionally, this study aims to determine the prevalence and anatomical distribution of musculoskeletal pain in this group.

## 2. Materials and Methods

### 2.1. Study Design

This was an observational study involving the administration of a questionnaire following participation in an educational workshop on the prevention of musculoskeletal injuries. The reporting of this observational study followed the STROBE guidelines [14]. The Research Ethics Committee of Pontevedra, Vigo, and Ourense (2025/085) approved the study protocol.

### 2.2. Participants

Participants were music students aged between 11 and 20 years who attended the educational workshop on injury prevention. The intervention was specifically targeted at string and wind instrument players, and workshops were conducted separately for each instrumental group.

To recruit participants and organize the workshops, the research team contacted the administrative boards of several professional conservatories located in the provinces of Pontevedra and Ourense. The activity was presented in advance, and a date was scheduled to allow interested students from the targeted instrumental families to enroll. The workshops were open to students at both elementary and professional levels. However, for elementary-level students, participation was recommended from the third year onwards, although this was not an exclusion criterion.

Inclusion criteria were music students who played a string or wind instrument, were aged between 11 and 20 years and provided informed consent. Exclusion criteria included cognitive difficulties that could impair the participants’ ability to understand or complete the questionnaire.

All participants were required to provide informed consent prior to inclusion in the study by completing the study questionnaire. For participants under 18 years of age, written informed consent had to be obtained from both parents or legal guardians, except in cases of single-parent families or when one parent provided consent on behalf of both.

### 2.3. Sample Size Calculation

All accessible participants who attended the training activities and met the selection criteria were included in the study. The resulting sample consisted of 60 students, a figure comparable to that used in previous observational studies of young musicians. Due to the exploratory nature of the study and the convenience sampling approach, no minimum sample size was established based on a superiority hypothesis.

### 2.4. Intervention and Measurement Instruments

The interventions were carried out between 2025 and 2026 and were conducted by a physical therapist specializing in the health of musicians. The aim of the workshop was to raise awareness of musculoskeletal injuries in musicians and to provide strategies to prevent their occurrence through changes in instrumental practice habits.

The number of participants per session was limited to a maximum of 20 musicians, and each workshop was tailored to a specific instrumental family: bowed string or wind instruments. The intervention was standardized and consisted of a workshop lasting approximately two hours.

During the first hour, participants were introduced to musculoskeletal injuries, their incidence, and the risk factors associated with their development. They were then taught warm-up and cool-down exercises to be performed before and after instrumental practice, respectively. Warm-up exercises included joint mobilization of the neck, shoulders, elbows, wrists, and hands, as well as mobilization of the diaphragm and jaw for wind musicians.

Cool-down exercises focused on stretching the key neck and upper-limb muscles involved in playing their instrument. Additionally, diaphragmatic and jaw relaxation techniques were included for wind musicians.

It should be noted that, prior to performing the exercises, postural correction was addressed using a self-elongation position, which participants were instructed to maintain throughout all the proposed exercises. The design of the exercises was based on prior research involving wind musicians [15].

In the second hour, participants received individualized guidance on how to improve and correct their posture and instrumental technique, based on direct observation while playing.

At the end of the session, participants were given a handout with the proposed exercises to practice at home and were invited to complete a questionnaire specifically designed for the activity.

The questionnaire was reviewed by a panel of six experts, including professionals from the fields of education, healthcare, and music. It included questions on sociodemographic characteristics (age and sex) as well as variables related to musical training, including the instrument played, the age at which instrumental practice began, and the participants’ level of musical education.

It also included questions related to musculoskeletal pain associated with instrumental practice, assessing the presence of pain at different time points (lifetime prevalence, during the last 12 months, last month, and last week), using the Musculoskeletal Pain Intensity and Interference Questionnaire [16], as well as its anatomical location using a body diagram.

Finally, the questionnaire included several items related to the workshop, assessed using a visual analog scale: satisfaction with the activity (“Did you like the workshop?”, from “not at all” to “very much”), perceived usefulness (“Do you think it will help your instrumental practice?”, from “not at all” to “very much”), intention to perform the proposed exercises (“Do you think you will perform any of the suggested exercises?”, from “none” to “all”), and whether the duration of the workshop was appropriate (yes/no).

Participants were also asked whether they were aware of the existence of musculoskeletal pain in musicians and, if so, how they had learned about it.

### 2.5. Statistical Analysis

Statistical analysis was performed using SPSS version 20.0 (IBM Corp., Armonk, NY, USA). Frequencies, percentages and 95% confidence intervals were calculated for qualitative variables, while means and standard deviations were computed for quantitative variables.

The Kolmogorov–Smirnov test was used to assess normality, revealing that the quantitative variables did not follow a normal distribution. The Mann–Whitney U test was used to compare workshop-related quantitative variables according to sex (male/female) and level of study (elementary/professional), and the Kruskal–Wallis test was used for comparisons across instrumental families (bowed strings/woodwind/brass). Effect size for Mann–Whitney U tests was calculated as r = Z/√N and interpreted as small (0.10), moderate (0.30), and large (0.50). For Kruskal–Wallis tests, epsilon squared (ε^2^) was calculated and interpreted as small (0.01), moderate (0.08), and large (0.26). Multivariate analysis was not performed due to the small sample size, the exploratory nature of the study, and the small number of participants in the individual instrumental subgroups. A 95% confidence level was established. A significance level of *p* < 0.05 was considered.

## 3. Results

The sample consisted of 60 participants, of whom 40 (66.7%) were female. The mean age was 14.4 ± 0.3 years. Regarding instrument type, 50 participants (83.3%) played bowed strings, while 10 played wind instruments, of whom 3 (5.0%) were woodwind players and 7 (11.7%) were brass players. Sample characteristics are presented in Table 1.

Regarding the workshop, 98.3% of participants reported that its duration was appropriate. When asked about their overall perception of the session, the mean score was 8.9 out of 10. Similarly, the question assessing whether the workshop could be helpful for their instrumental practice yielded a mean score of 8.9 out of 10. In addition, participants reported a mean score of 7.9 out of 10 regarding their intention to perform the proposed exercises, where a score of 10 indicated the intention to perform all exercises. No statistically significant differences were found when comparing these variables according to sex, instrument family, or level of study (*p* > 0.05). Moreover, the observed effect sizes were negligible across all comparisons (Table 2).

Regarding the presence of pain, 35 participants (58.3%) reported experiencing musculoskeletal pain at some point in their lifetime. Pain was reported by more than half of the musicians during the previous 12 months (*n* = 33, 55.0%), by 31.7% (*n* = 19) during the past month, and by 20.0% (*n* = 12) during the past week.

In terms of anatomical distribution, the most frequently reported pain locations were the left shoulder (45.0%), the left wrist/hand (40.0%), the neck (35.0%), and the right shoulder (33.3%) (Table 3). Among those reporting head pain, five participants specified mandibular pain. Regarding the lower limbs, only two musicians reported knee pain, which was bilateral in both cases.

**Figure 1:**
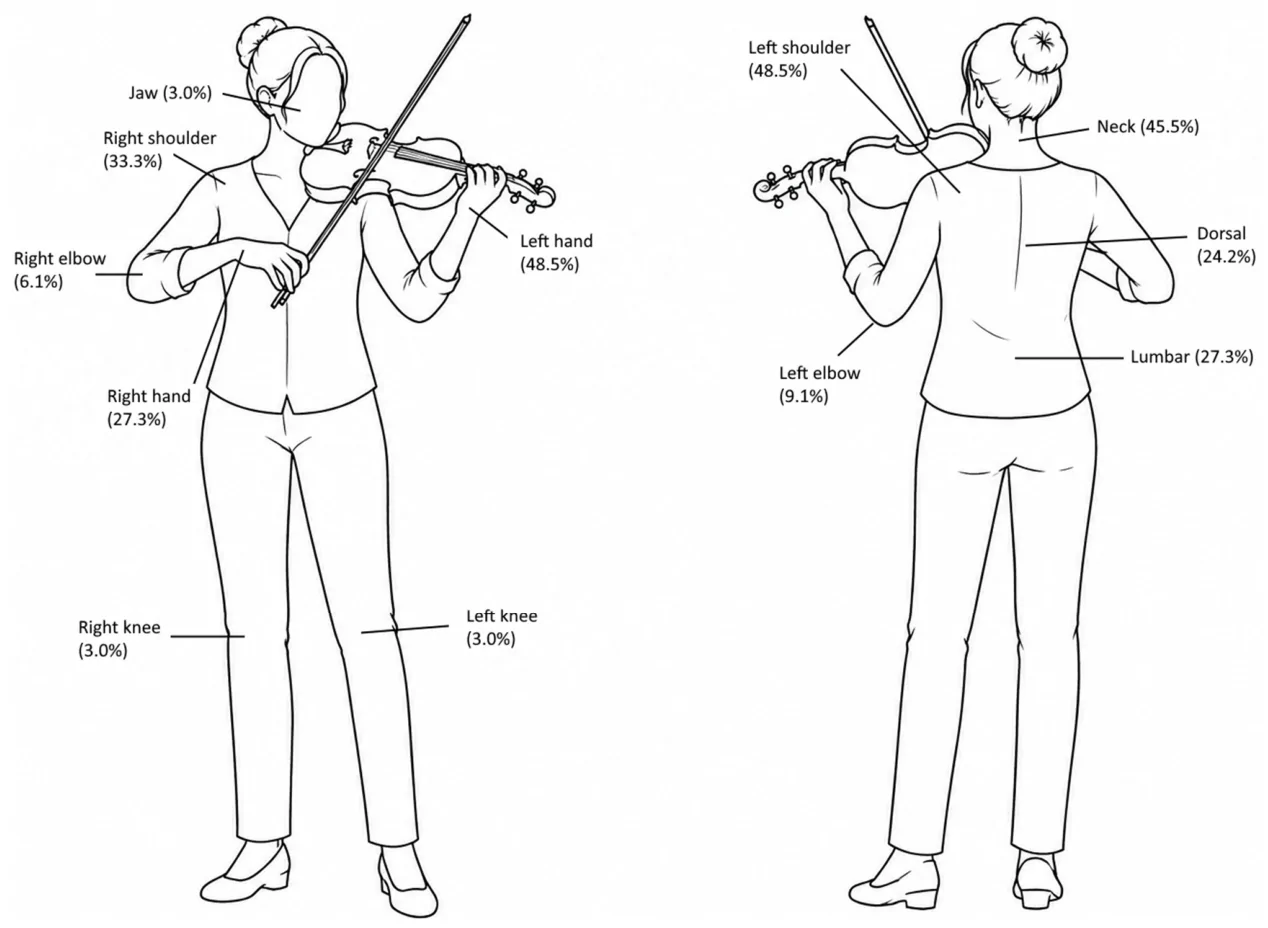
Prevalence of pain by anatomical regions in violinists.

Due to the higher number of violinists in the sample, the distribution of pain locations was further analyzed within this subgroup (Figure 1), showing that the left shoulder and left wrist/hand (48.5%), followed by the neck (45.5%), were the most frequently affected regions.

Finally, when participants were asked about their awareness of musculoskeletal injuries in musicians, 52 (86.7%) reported being aware of them. Among these participants, 36 indicated that this knowledge had been acquired from their instrument teacher, 15 from healthcare professionals, 13 from family members, 9 through social media, and 7 from other sources (Table 4).

## 4. Discussion

Satisfaction with the workshop for the prevention of musculoskeletal injuries in young music students was high, with a large proportion of participants indicating their willingness to adopt the exercises and behavioral changes proposed during the session. In this regard, Ioannou and Altenmüller [17] reported that a substantial percentage of music students considered it necessary to incorporate anatomy and physiology courses into music education programs. Additionally, more than half of the participants reported having experienced musculoskeletal pain related to instrumental practice at some point in their lives, a finding consistent with previous studies [5].

The high prevalence of health problems associated with musical practice highlights the need to integrate anatomy, physiology, and health education into music curricula. Recent studies recommend including occupational health training for musicians, as it contributes to improved body awareness, promotes the modification of risk-related behaviors, and reduces the incidence of pain and injuries [18,19]. Furthermore, the physical and psychological vulnerability of musicians supports the implementation of educational programs focused on self-care and injury prevention from an early stage [20]. Overall, these findings reinforce the importance of incorporating specific training on human anatomy and physiology as an essential component of music education.

Due to this high prevalence, it is essential to take action to reduce the incidence of musculoskeletal injuries and their impact on musicians. Beyond the pain they cause, such conditions may lead to a reduction in practice time and even result in time away from work or study [21,22,23]. However, only 26% of instrumentalists who experienced playing-related musculoskeletal disorders reported full recovery [21]. Consequently, feelings of fear and apprehension are commonly described by musicians following such injuries [24]. Notably, most music students do not believe they can engage in instrumental practice without experiencing physical limitations [25]. Furthermore, it has been reported that 13% of musicians experience pain consistently during practice [17].

Given these findings and considering that prevention is preferable to treatment [26], it is important to incorporate preventive strategies at all levels of music education [17,27]. In this regard, musicians should be educated about the existence of musculoskeletal disorders and provided with practical strategies to prevent them. These include performing warm-up exercises away from the instrument before practice and cool-down exercises afterward. Additionally, identifying and addressing individual issues related to posture and instrumental technique may represent effective preventive strategies. In doing so, the main risk factors identified by musicians as contributing to the development of musculoskeletal injuries are directly addressed [28].

On the other hand, the results of the present study indicate high level of satisfaction among the participating musicians with the workshops. Participants reported that the sessions could meaningfully support their instrumental practice, that they were enjoyable, and that their duration was appropriate. Moreover, most participants expressed their intention to perform the exercises introduced during the sessions. It would be valuable to assess the outcomes of their long-term implementation, as the absence of perceived benefits in performance or overall experience may significantly reduce adherence to an intervention [29].

One of the main factors influencing adherence to an exercise program is the perception that it is important for instrumental practice and for the prevention of future health problems [15]. In addition, the workshop included a simple, short-duration exercise program designed to facilitate adherence, as lack of time is one of the main reasons for not following an exercise program [30,31].

In our study, teachers were identified as the primary source of information regarding musculoskeletal injuries, consistent with findings reported among non-graduate musicians in the study by Stanek et al. [32], followed by healthcare professionals. Ioannou and Altenmüller [17] reported that 77% of music students believe that music schools should provide access to a specialized physician to address musculoskeletal problems related to instrumental practice. In this context, pain is the most common symptom prompting musicians to seek medical consultation [26,33], with musculoskeletal disorders being the most frequently reported condition [2,5,33].

Furthermore, the most common pain locations identified among the young musicians in our study were the shoulders, left hand, and neck. These findings are consistent with those reported in previous studies on musicians [34,35,36,37]. Specifically, among violinists, the shoulders, neck, and distal region of the left upper limb were particularly affected, in line with other studies [22,38,39]. In this respect, scapular dyskinesis observed in violinists [40] may contribute to the occurrence of pain in the neck and shoulder regions during instrumental practice.

Several limitations were identified in this study. A notable limitation of this study was the use of an ad hoc, non-validated questionnaire to assess both the participants’ satisfaction and the presence of musculoskeletal pain. Given the descriptive and observational nature of this research, the instrument was specifically designed to capture the unique components and objectives of the targeted educational workshop developed by young string and wind musicians. In addition, the absence of a control group and the lack of pre- and post-intervention measurements constitute additional limitations. Although the overall sample size was sufficient for the study objectives, it was not possible to draw instrument-specific conclusions—except in the case of violinists—due to the limited number of participants within each instrumental group. Additionally, the lack of follow-up regarding the performance of the proposed exercises and the implementation of recommended changes precluded an evaluation of their effectiveness, as well as long-term satisfaction and adherence.

It should also be noted that participation was voluntary, which may have introduced self-selection bias. Students with a greater interest in musicians’ health or injury prevention may have been more likely to attend the workshop and participate in the study. Furthermore, the high ratings observed for satisfaction and intention to perform the proposed exercises may have been influenced by social desirability bias, as participants may have tended to provide more favorable responses.

Another important limitation is that the study assessed participants’ satisfaction immediately after the workshop, without examining whether the intervention produced changes in relevant outcomes, such as knowledge, behavior, exercise adherence, or symptoms. Accordingly, the findings support the acceptability of the workshop but do not allow conclusions to be drawn regarding its effectiveness.

The workshop incorporated both a general educational session and individualized postural and technical advice. As these components were delivered concurrently, it was not possible to determine their independent contributions to participants’ satisfaction or perceived usefulness.

From a clinical perspective, interventions aimed at raising awareness of common musculoskeletal problems among musicians represent a key tool for highlighting their importance and promoting effective prevention strategies. Furthermore, increasing awareness of the role of physiotherapy in this field may facilitate recognition of physical therapists as appropriate healthcare professionals for both the prevention and treatment of these disorders.

Future research should consider the psychometric validation of this tool or the integration of standardized instruments to strengthen the comparability of the findings. Future study designs should incorporate a pre-intervention assessment to establish participants’ baseline knowledge, awareness, and preventive behaviors. Such baseline measurements are essential to determine whether the workshop produces meaningful changes in participants’ understanding of and attitudes towards injury prevention. Additionally, future studies should consider designs that allow the independent effects of the educational workshop and the individualized technical advice to be evaluated separately.

Likewise, it would be worthwhile to examine whether participation in this type of educational intervention leads to actual changes in the habits of young musicians. Furthermore, it would be valuable to assess, over both the short and long term, the effects of health education on physical outcomes and instrumental practice behaviors.

## 5. Conclusions

The participating musicians reported a high level of satisfaction with the workshop, considering it both engaging and relevant to their instrumental practice. Additionally, a large proportion of participants expressed their intention to incorporate the proposed exercises. These findings support the acceptability and perceived usefulness of the workshop; however, they should not be interpreted as evidence of actual improvements in preventive behaviors and exercise adherence or reductions in musculoskeletal injuries or symptoms.

Regarding the prevalence of musculoskeletal pain, it was found to be high both over participants’ lifetimes and at recent time points. The most frequently affected areas were the shoulders, the cervical spine, and the left wrist–hand region.

However, these findings should be interpreted with caution due to the study’s limitations, particularly the voluntary nature of participation, the possibility of self-selection and social desirability biases, and the cross-sectional design, which precludes establishing causal relationships or evaluating long-term effects.

## Figures and Tables

**Table 1:** Sample characteristics.

Variable	Mean ± SD	Median	IQR 25–75
Age (years)	14.4 ± 0.28	14	13–16
Instrument starting age (years)	7.6 ± 0.15	8	7–8
Practice (days/week)	4.7 ± 0.19	5	3–6
**Instrument Family**	**Instrument**	***n* (%)**	
Strings	Violin	33 (55.0)	
	Viola	6 (10.0)	
	Cello	6 (10.0)	
	Double bass	5 (8.3)	
Woodwinds	Clarinet	1 (1.7)	
	Bassoon	1 (1.7)	
	Saxophone	1 (1.7)	
Brass	Trombone	4 (6.7)	
	French horn	3 (5.0)	
**Educational Level**	**Course**	***n* (%)**	
Elementary	1st	0 (0.0)	
	2nd	3 (5.0)	
	3rd	6 (10.0)	
	4th	3 (5.0)	
Professional	1st	7 (11.7)	
	2nd	9 (15.0)	
	3rd	9 (15.0)	
	4th	9 (15.0)	
	5th	6 (10.0)	
	6th	8 (13.3)	

IQR: interquartile range; SD: standard deviation.

**Table 2:** Participants’ perception of the workshop by groups.

Variable	Category	Liking Median (IQR)	Statistical Test	*p*-Value	Effect Size
Sex	Male	9.8 (7.5–10.0)	U = 373.0	0.648	0.059
	Female	10.0 (7.8–10.0)			
Instrument Family	Bowed strings	10.0 (7.5–10.0)	H = 0.858	0.651	0.000
	Woodwinds	10.0 (9.5–10.0)			
	Brass	9.5 (8.2–10.0)			
Educational Level	Elementary	9.6 (6.9–10.0)	U = 256.0	0.524	0.082
	Professional	10.0 (7.6–10.0)			
**Variable**	**Category**	**Helpfulness Median (IQR)**	**Statistical test**	***p*** **-Value**	**Effect size**
Sex	Male	9.8 (7.5–10.0)	U = 387.0	0.827	0.028
	Female	9.9 (8.1–10.0)			
Instrument Family	Bowed strings	10.0 (7.8–10.0)	H = 1.383	0.501	0.000
	Woodwinds	10.0 (8.0–10.0)			
	Brass	8.7 (8.5–9.7)			
Educational Level	Elementary	10.0 (8.7–10.0)	U = 232.0	0.268	0.143
	Professional	9.5 (7.9–10.0)			

IQR: interquartile range; H: Kruskal–Wallis test; U: Mann–Whitney U test.

**Table 3:** Presence of pain by body region.

Body Region	*n* (%)	95% CI		
Neck	21 (35.0)	0.23–0.48		
Thoracic	15 (25.0)	0.15–0.38		
Lumbar	13 (21.7)	0.12–0.34		
Head/Jaw	6 (10.0)	0.04–0.20		
	**Right *n* (%)**	**95% CI**	**Left *n* (%)**	**95% CI**
Shoulder/Arm	20 (33.3)	0.22–0.47	27 (45.0)	0.32–0.58
Elbow/Forearm	4 (6.7)	0.02–0.16	4 (6.7)	0.02–0.16
Wrist/Hand/Fingers	16 (26.7)	0.16–0.40	24 (40.0)	0.28–0.54
Hip/Thigh	0 (0.0)	0.00–0.06	0 (0.0)	0.00–0.06
Knee/Calf	2 (3.3)	0.00–0.12	2 (3.3)	0.00–0.12
Ankle/Foot	0 (0.0)	0.00–0.06	0 (0.0)	0.00–0.06

CI: confidence interval.

**Table 4:** Sources of information of musculoskeletal injuries.

Source of Information	*n* (%)	95% CI
Awareness of musculoskeletal injuries	22 (86.7)	0.75–0.94
- Healthcare professionals (physicians, physiotherapists, etc.)	15 (25.0)	0.15–0.38
- Music teacher	36 (60.0)	0.47–0.72
- Family	13 (21.7)	0.12–0.34
- Social media	9 (15.0)	0.07–0.27
- Other sources:	7 (11.7)	0.05–0.23
Friends	3 (5.0)	
Personal experience	3 (5.0)	
Lecture/workshop	1 (1.7)	

CI: confidence interval.

## Data Availability

The data presented in this study are available on request from the corresponding author due to the inclusion of children in the sample and as a precautionary measure to preserve participant anonymity, given that the article identifies the centers where the study was conducted.

## References

[1] Backiev L., Bastepe-Gray S., Mueller D., Watson M., Chiang C.C., Emam M., Lasner A.N. (2024). Updates in performing arts medicine: A clinical overview for instrumental musicians and dancers. Curr. Phys. Med. Rehabil. Rep..

[2] Autret L., Shipley É., Lodde B., Hinojosa-Azaola A., Saraux A. (2025). Rheumatic and musculoskeletal disorders in musicians: Risks, adaptations and management. RMD Open.

[3] Cardoso M., Leonido L., Morgado E. (2025). Neuromuscular control and musculoskeletal injuries in musicians: A critical review. Motricidade.

[4] García Gómez M. (2018). Las enfermedades profesionales de los músicos, el precio de la perfección. Arch. Prev. Riesgos Labor..

[5] Cerda-Díaz L., Antúnez-Riveros M., Bracchiglione J., Opazo-Campusano R., Núñez-Cortés R. (2026). Musculoskeletal disorders in musicians: An overview of systematic reviews. Occup. Med..

[6] Rodríguez-Gude C., Pino-Juste M. (2021). Análisis de los planes de estudios de los Conservatorios superiores en España en relación a la prevención de lesiones. Desempeño Docente y Formación en Competencia Digital en la Era SARS COV 2.

[7] Council of Arts Accrediting Associations (1991). An Overview of Health Issues for Performing and Visual Arts Students. https://nasad.arts-accredit.org/wp-content/uploads/sites/3/2016/03/CAAA-Health_Issues-2009.pdf.

[8] Chesky K., Dawson W., Manchester R. (2006). Health promotion in schools of music: Initial recommendations for schools of music. Med. Probl. Perform. Artists.

[9] Su M.-J., Su Y.-H., Lin Y.-J., Chen H.-S. E-learning development on health promotion for music performers in Taiwan. Proceedings of the 2011 IEEE 13th International Conference on e-Health Networking, Applications and Services.

[10] Consejo General de Colegios de Fisioterapeutas de España (2018). Resolución No 1/2018 Por La Que Se Aprueba la Definición del Acto Fisioterápico. [Resolution No. 1/2018 on the Definition of the Physiotherapy Act]. https://www.boe.es/buscar/doc.php?id=BOE-B-2018-49464.

[11] Belando-Pedreño N., Blanco-García M.E., Chamorro J.L., García-Martí C. (2023). Pilot Study on Satisfaction in Children and Adolescents after a Comprehensive Educational Program on Healthy Habits. Nutrients.

[12] Nagy-Pénzes G., Vincze F., Bíró É. (2022). A School Intervention’s Impact on Adolescents’ Health-Related Knowledge and Behavior. Front. Public Health.

[13] Pérez-Jorge D., González-Luis M.A., Rodríguez-Jiménez M.d.C., Ariño-Mateo E. (2021). Educational Programs for the Promotion of Health at School: A Systematic Review. Int. J. Environ. Res. Public Health.

[14] von Elm E., Altman D.G., Egger M., Pocock S.J., Gøtzsche P.C., Vandenbroucke J.P. (2014). The Strengthening the Reporting of Observational Studies in Epidemiology (STROBE) Statement: Guidelines for reporting observational studies. Int. J. Surg..

[15] Rodríguez-Gude C., Taboada-Iglesias Y., Pino-Juste M. (2025). Design and assessment of a musculoskeletal injury prevention programme for wind players. Work.

[16] Berque P., Gray H., McFadyen A. (2014). Development and psychometric evaluation of the Musculoskeletal Pain Intensity and Interference Questionnaire for professional orchestra Musicians. Man. Ther..

[17] Ioannou C.I., Altenmüller E. (2015). Approaches to and treatment strategies for playing-related pain problems among Czech instrumental music students: An epidemiological study. Med. Probl. Perform. Artist.

[18] Evans A., Rennie-Salonen B., Wijsman S., Ackermann B. (2024). A scoping review of occupational health education programs for music students and teachers. Res. Stud. Music. Educ..

[19] Matei R., Broad S., Goldbart J., Ginsborg J. (2018). Health Education for Musicians. Front. Psychol..

[20] Shoebridge A., Osborne M.S. (2025). Wellbeing for young elite musicians: Development of a health protocol from a student perspective. Front. Psychol..

[21] Kenny D.T., Driscoll T., Ackermann B.J. (2018). Effects of aging on musical performance in professional orchestral musicians. Med. Probl. Perform. Artist.

[22] Kochem F.B., Silva J.G. (2017). Prevalence and associated factors of playing-related musculoskeletal disorders in Brazilian violin players. Med. Probl. Perform. Artist.

[23] Steemers S., van Middelkoop M., de Boks G.G., van Rijn R.M., Bierma-Zeinstra S.M.A., Stubbe J.H. (2020). The impact of injury definitions on measures of injury occurrence in classical music students: A prospective cohort study. BMC Musculoskelet. Disord..

[24] Guptill C.A. (2011). The lived experience of professional musicians with playing-related injuries: A phenomenological inquiry. Med. Probl. Perform. Artist.

[25] Steinmetz A., Möller H., Seidel W., Rigotti T. (2012). Playing-related musculoskeletal disorders in music students-associated musculoskeletal signs. Eur. J. Phys. Rehabil. Med..

[26] Brandfonbrener A.G. (2003). Musculoskeletal problems of instrumental musicians. Hand Clin..

[27] Silva A.G., Lã F.M., Afreixo V. (2015). Pain prevalence in instrumental musicians: A systematic review. Med. Probl. Perform. Artist.

[28] Rodríguez Gude C., Moro López Menchero P., Rodríguez Rodríguez L., Carrillo Carbajal O.Á., Hernández Lucas P., Guedes Lobato R.J., Asociación Universitaria de Educación y Psicología—ASUNIVEP (2026). Factores de riesgo y hábitos en la práctica instrumental relacionados con la aparición de lesiones musculoesqueléticas en músicos/as. Educación e Investigación en Salud: Claves para la Innovación.

[29] McCrary J.M., Halaki M., Sorkin E., Ackermann B.J. (2016). Acute warm-up effects in submaximal athletes: An EMG study of skilled violinists. Med. Sci. Sports Exerc..

[30] Ajidahun A.T., Myezwa H., Mudzi W., Wood W.A. (2019). Barriers and facilitators in implementing an exercise-based injury prevention program for string players. Work.

[31] Seitz M.W., Levy J.J., Murphy B.A. (2020). Examining college music majors’ perceived barriers and motivation to exercise. Med. Probl. Perform. Artist.

[32] Stanek J.L., Komes K.D., Murdock F.A. (2017). A cross-sectional study of pain among U.S. college music students and faculty. Med. Probl. Perform. Artist.

[33] Lederman R. (2003). Neuromuscular and musculoskeletal problems in instrumental musicians. Muscle Nerve.

[34] Viljamaa K., Liira J., Kaakkola S., Savolainen A. (2017). Musculoskeletal Symptoms Among Finnish Professional Orchestra Musicians. Med. Probl. Perform. Artist.

[35] Steinmetz A., Scheffer I., Esmer E., Delank K.S., Peroz I. (2015). Frequency, severity and predictors of playing-related musculoskeletal pain in professional orchestral musicians in Germany. Clin. Rheumatol..

[36] Vastamäki M., Ristolainen L., Heliövaara M., Vastamäki H. (2020). Musculoskeletal pain among Finnish orchestra musicians versus core workforce. Occup. Med..

[37] Rodríguez-Gude C., Taboada-Iglesias Y., Pino-Juste M. (2025). Location of Musculoskeletal Pain in Musicians According to Gender, Academic Status and Instrument. Muscles Ligaments Tendons J..

[38] Stach B., Bieńko E., Pilch A., Jekiełek M. (2026). Assessment of pain and postural defects among young adults playing the violin. Med. Pr..

[39] Cesar Santos A., Kochem F.B., Crepaldi Lunkes L. (2025). Relationship Between Musculoskeletal Pain, Psychosocial Aspects, and Quality of Work Life in Violinists and Violists: A Cross-Sectional Study in Rio de Janeiro, Brazil. Med. Probl. Perform. Artist.

[40] Tawde P., Dabadghav R., Bedekar N., Shyam A., Sancheti P. (2016). Assessment of cervical range of motion, cervical core strength and scapular dyskinesia in violin players. Int. J. Occup. Saf. Ergon..

